# Comprehensive compilation and quality assessment of street-level urban air temperature measurements across European networks

**DOI:** 10.1038/s41597-026-06804-4

**Published:** 2026-02-14

**Authors:** Setareh Amini, Adrian Huerta, Jörg Franke, Yuri Brugnara, Steven Caluwaerts, Julien Anet, Stevan Savić, Moritz Gubler, Gert-Jan Steeneveld, Lee Chapman, Fred Meier, Vincent Dubreuil, Andreas Christen, Matthias Zeeman, Branislava Lalić, Sebastian Schlögl, Jukka Käyhkö, AmirMasoud Azadfar, Stefan Brönnimann

**Affiliations:** 1 Oeschger Centre for Climate Change Research (OCCR), University of Bern, Bern, Switzerland; 2 Institute of Geography, University of Bern, Bern, Switzerland; 3 Department of Physics and Astronomy, Ghent University, Krijgslaan 281, 9000 Gent, Belgium; 4 Meteorological Institute of Belgium, Ringlaan 3, 1180 Ukkel, Belgium; 5 Umwelt- und Gesundheitsschutz (UGZ), Fachbereich Stadtklima, Zürich, Switzerland; 6 Faculty of Sciences, University of Novi Sad, Trg Dositeja Obradovica 3, 21000 Novi Sad, Serbia; 7 Faculty of Natural Sciences and Mathematics, University of Banja Luka, Mladena Stojanovica 2, 78000 Banja Luka, Bosnia and Herzegovina; 8 Institute for Lower Secondary Education, Bern University of Teacher Education, Bern, Switzerland; 9 Wageningen University, Meteorology and Air Quality Section, Wageningen, The Netherlands; 10 Iniversity of Birmingham, Edgbaston, Birmingham B15 2TT UK; 11 Institue of Ecology, Technische Universität Berlin, Berlin, Germany; 12 LETG-UMR 6554, CNRS, University of Rennes 2, F-35000 Rennes, France; 13 Albert-Ludwigs-Universität Freiburg, Environmental Meteorology, Freiburg, Germany; 14 Faculty of Agriculture, University of Novi Sad, Novi Sad, Serbia; 15 meteoblue AG, Basel, Switzerland; 16 University of Turku, Department of Geography and Geology, Turku, Finland; 17 Department of Computer Science, Lakehead University, Thunder Bay, Canada

**Keywords:** Climate sciences, Environmental impact, Natural hazards, Climate and Earth system modelling

## Abstract

This study provides a comprehensive dataset (FAIRUrbTemp) that addresses the lack of high-resolution urban air temperature data across Europe. It compiles sub-hourly street-level air temperature data from 811 low-cost to commercial sensors across several European cities and offers data in a quality-controlled, standardized format in sub-hourly, hourly, and daily resolutions. In addition, detailed metadata, as an important source of information in urban studies, is provided at network, station, and measurement levels. This pan-European dataset is rigorously quality-controlled using a serially automatic method applicable to diverse city-scale air temperature data, which identifies systematic and minor inconsistencies to enhance reliability. Expert-based validation shows that the QC reliably identifies problematic measurements, while its performance varies across urban and climatic settings due to local environmental and instrumental effects. To ensure transparency, the results of the quality control are provided to the user together with the original value in the dataset. The validated FAIRUrbTemp is a valuable resource for urban climate studies, with direct applications in validating microclimate models, assessing heat-health risks, and informing climate-adaptive urban planning.

## Background & Summary

Near-surface air temperature is a critical climatological variable with significant impacts in various domains, including human health^1,2^, economy^3,4^ and society^5^. Changes in frequency, intensity, spatial extent, and duration of extreme events such as recent severe European summer heatwaves and drought^6,7^ have significantly impacted human, flora and fauna life. Heatwaves are exacerbated in cities due to impervious surfaces, buildings, and other factrors, leading to locally elevated temperatures known as urban heat island effect^8^. Due to the high population density and thus high exposure, including vulnerable persons, heatwaves pose a significant risk to many cities in Central Europe and elsewhere. To design effective mitigation and adaptation strategies and reduce future risks, it is essential to gather information on micro-climate patterns in cities, reflecting urban-rural differences but also intra-urban variability^9^.

Consequently, reliable and accurate near-surface air temperature measurements in cities are crucial. Due to their high installation and maintenance costs, as well as required site setting^10^, professional weather stations are often difficult to install in urban areas^11^. Fortunately, recent affordable, compact environmental sensors can now regularly achieve sub-hour sampling intervals and temperature accuracy of ±0.5^°^C (with precision down to 0.1°C). Such performance is adequate to resolve intra-urban temperature gradients for heat island mapping and local trend analysis, while applications such as high-precision model evaluation may require even smaller errors^12–16^.

In Europe, significant efforts have led to the development of Climate Services, providing data from ground-based measurements, satellite data, and weather and climate simulations, and ensuring these datasets are publicly accessible through initiatives such as C3S (Copernicus Climate Change Service). These established data repositories are extensively utilized across research, education, and economic sectors. However, a notable deficiency in these repositories is the absence of micrometeorological data from cities, or in the case of the presence in the global products, the accuracy of the data is very low and is not a reliable source for microclimate studies^17^. Within urban areas, such data are essential for urban planners, environmental scientists, and policymakers actively involved in crafting strategies for climate-resilient urban planning^18^.

To address this gap and to provide this type of data, in the framework of the COST Action CA20108 FAIRNESS (FAIR NEtwork of micrometeorological measurements) project the Fair Micromet Portal - FMP 2.0, (available at: https://www.fairnessca20108.eu/micromet_ksp/) was developed. The main goal of FMP 2.0 is to improve the Findability, Accessibility, Interoperability and Reusability (FAIR)^19^ character of micrometeorological data and to publish: a) a compiled inventory of available and quality proven micrometeorological in situ data sets on the European level and beyond, b) a structured guidance framework for FAIRification of micrometeorological data, and c) examples of rural and urban FAIR data sets. The initiative focuses on standardizing data quality, filling data gaps, offering detailed metadata descriptions, and making FAIR datasets accessible for rural and urban areas^18^.

Building on the Cost Action FAIRNESS project, this research systematically identified and compiled a temperature dataset from 12 European urban networks. This data was subsequently processed into a common format before being quality controlled. This process was designed to capture issues such as calibration errors, systematic biases, drifts, unsuitable station configurations or locations, inadequate maintenance of the stations, communication, and software errors that produce erroneous or missing data^20^. For this purpose, a tailored quality control (QC) procedure was developed by adapting existing strategies (e.g., Hunziker *et al*.^21^) to the studied urban climate networks. As an additional application of quality assurance best practices, metadata is also collected and provided within a single dataset. According to the Global Climate Observing System monitoring principles^22^, metadata, which includes the specifics and history of local conditions, instruments, operational procedures, and data processing algorithms, should be compiled and maintained with the same care as the measurements themselves. Metadata is crucial for FAIR principles and also for facilitating precise interpretation and analysis of longer-term datasets, as it allows detecting, explaining and correcting inconsistencies. Metadata is therefore essential for managing urban temperature networks^23–25^. In addition, data-metadata inconsistencies will become increasingly challenging when studying more than one network, which is why the main goal of this study is to organize and standardize all the network information into a homogeneous format.

This paper introduces the FAIRUrbTemp dataset, a high-resolution, open-access collection of near-surface air temperature data from low-cost and commercial street-level sensors across 12 European cities. Designed to address the persistent lack of spatially dense and harmonized temperature data in urban areas, the dataset captures conditions within the urban canopy layer at sub-hourly, hourly, and daily resolutions. It is provided in a standardised format with detailed metadata. By leveraging cost-effective measurement technologies, FAIRUrbTemp significantly expands the potential for fine-scale climate monitoring and analysis across diverse urban environments. Its applications span a wide range of research areas, including the investigation of intra-urban temperature variability, evaluation of urban heat island intensity, calibration and validation of weather and climate models, assessment of heat-related health risks, and the development of evidence-based strategies for climate-resilient urban planning.

## Methods

### Overview

**Fig. 1:**
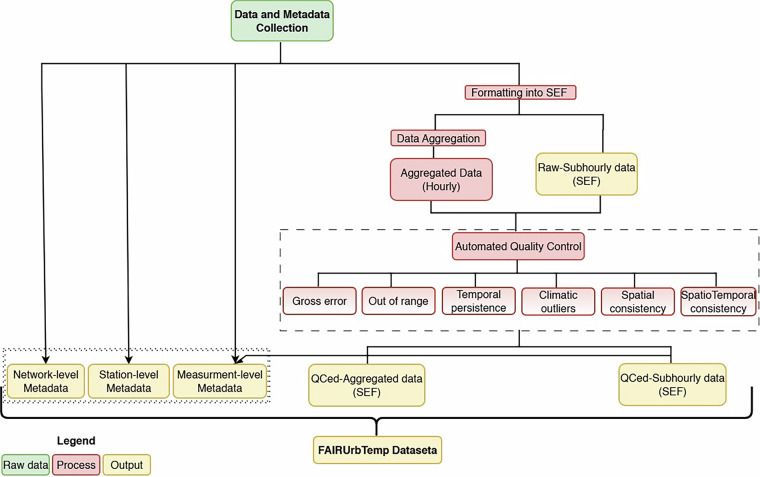
Schematic overview of the near-surface air temperature data collection and generation of the open access dataset. Raw data, related processes, and main output files are specified.

The process of generating the dataset is illustrated in Fig. 1. First, subhourly air temperature data were collated from existing street-level weather station networks within the COST action FAIRNESS project. Second, we converted all data into the Station Exchange Format (SEF)^26^. SEF files contain both data and metadata. Third, the QC procedure was applied (see sect. “Quality Control”). Finally, we aggregated the data into hourly mean, daily max, and daily min. The processed data were also stored in SEF format after QC.

### Data

#### Compilation of existing data

**Fig. 2:**
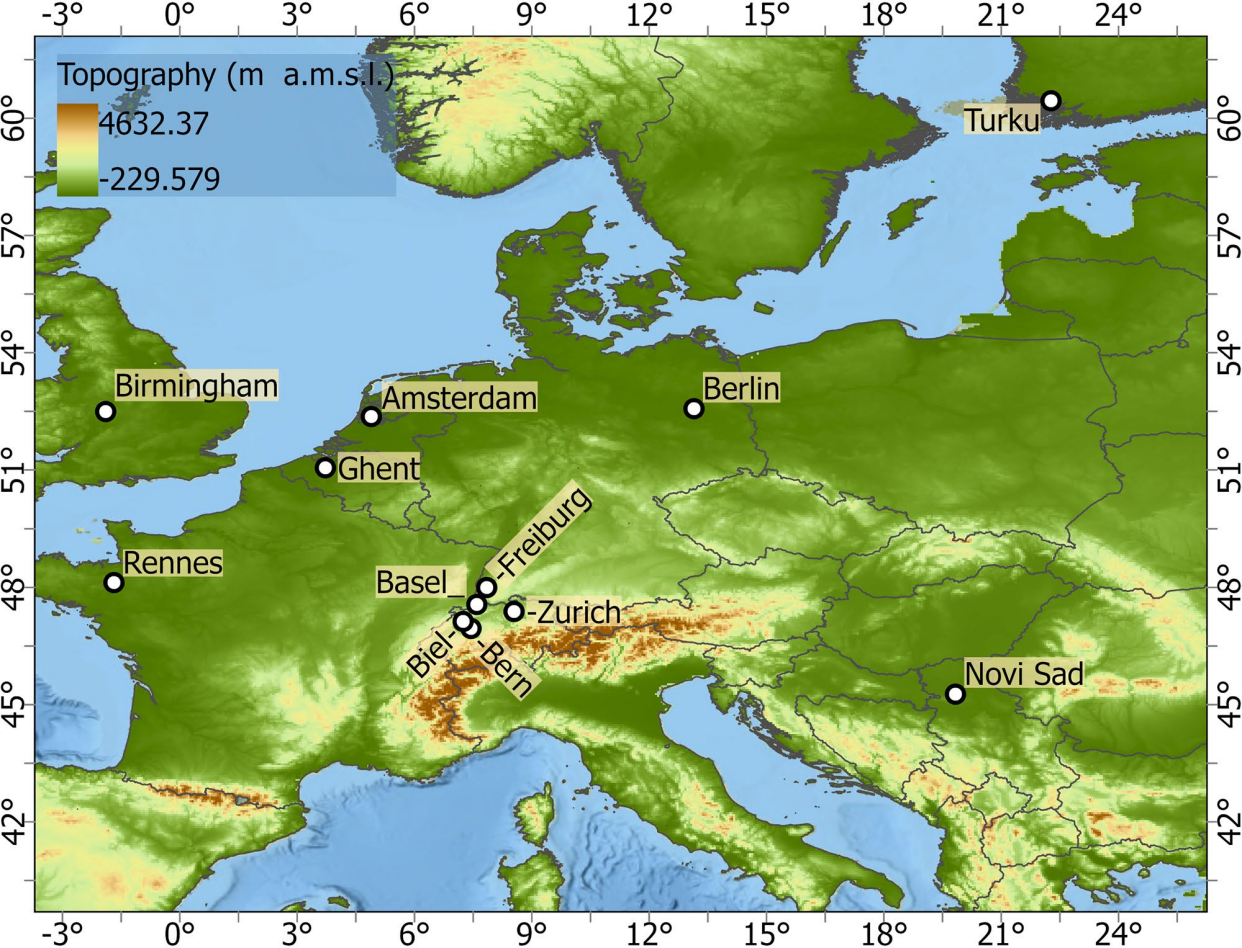
Geographical distribution of the studied cities in Europe. With the main topography of Europe. The DEM is an SRTM 30 m. The dots indicate the locations of the European networks evaluated in this research. Each network has a specific number of stations.

**Fig. 3:**
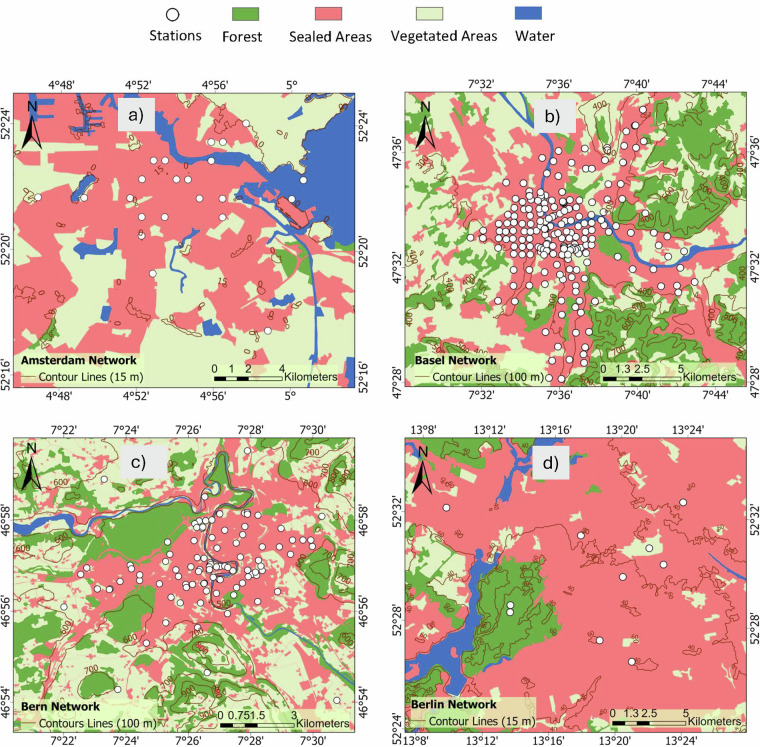
Locations of weather stations (**a**) Amsterdam, (**b**) Basel, (**c**) Bern and (**d**) Berlin, in relation to land cover and topography (Urban Atlas; EEA, 2018)(part 1 of 3).

**Fig. 4:**
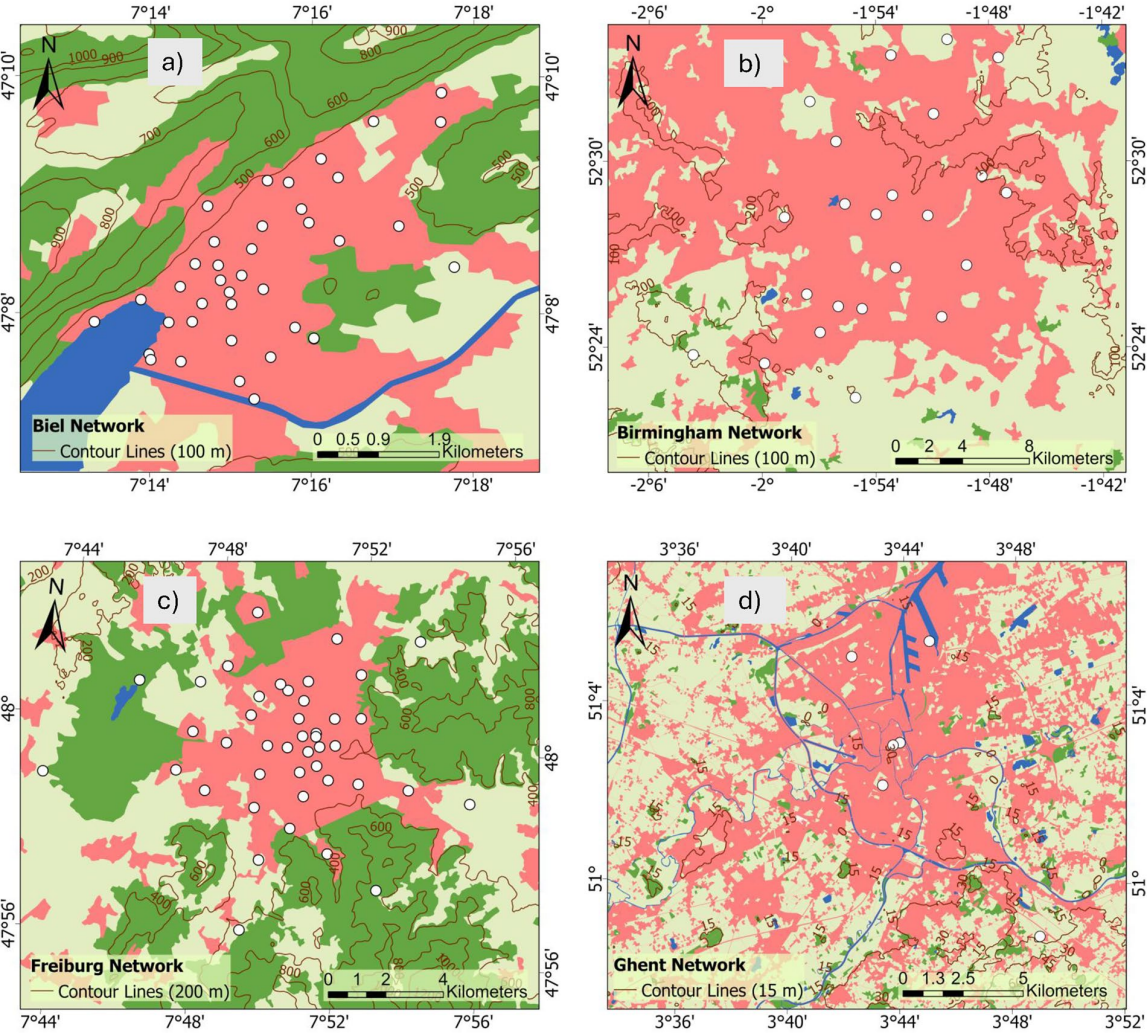
Locations of weather stations (**a**) Biel, (**b**) Birmingham, (**c**) Freiburg and (**d**) Ghent, in relation to land cover and topography(Urban Atlas; EEA, 2018) (part 2 of 3).

**Fig. 5:**
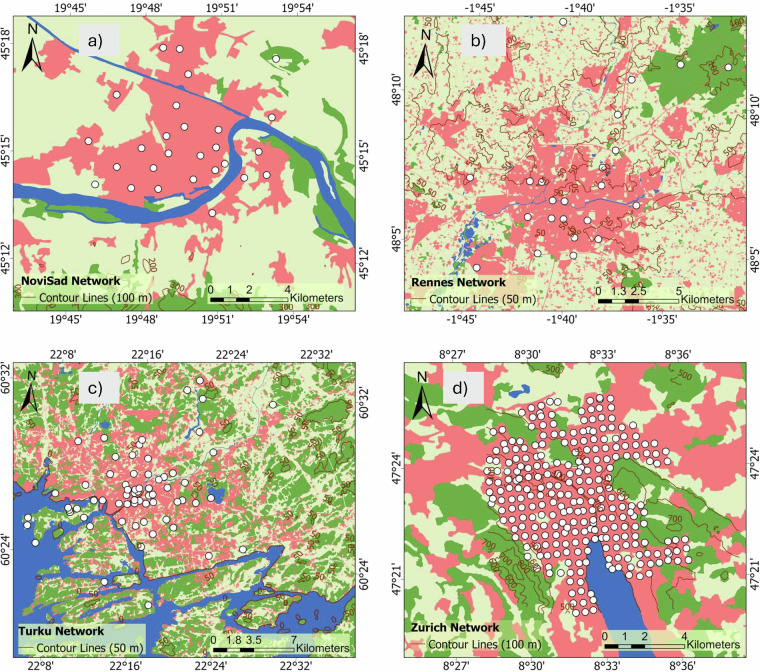
Locations of weather stations (**a**) Novi Sad, (**b**) Rennes, (**c**) Turku and (**d**) Zurich, in relation to land cover and topography(Urban Atlas; EEA, 2018) (part 3 of 3).

**Fig. 6:**
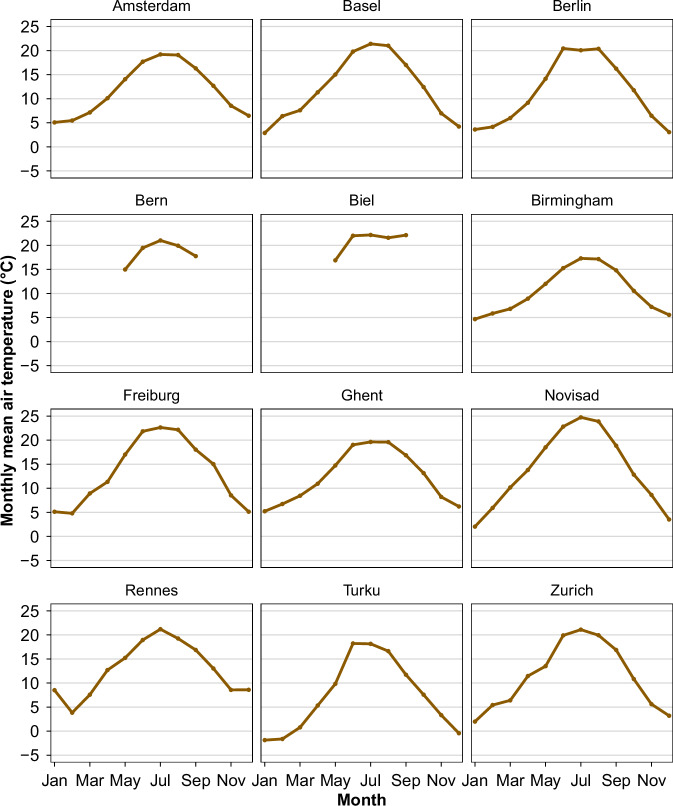
Monthly mean air temperature climatology for each studied network, calculated as the mean across all available stations within a network and averaged over the respective study period of each network. Because the observation periods differ between networks, the climatologies shown represent network-specific time averages rather than a common reference period.

Data were compiled from 12 established European networks active within the broader COST action. The networks are Bern (Gubler *et al*.^15^, Dataset^27^), Biel (Biel (T.M. Erismann *et al*.^28^), Basel (Schlögl *et al*.^29^), Amsterdam (Ronda *et al*.^30^), Birmingham (Muller *et al*.^11^, Dataset^31^), Freiburg (Plein *et al*.^32^, Feigel *et al*.^33^, Dataset^34^), Ghent (Caluwaerts *et al*.^35^), Berlin (Fenner *et al*.^36^, Dataset^37^), Novi Sad (Šećerov *et al*.^38^, Dataset^39^), Rennes (Dubreuil *et al*.^40^, Dataset^41^), Turku (TURCLIM, Alvi *et al*.^42^), and Zurich (Anet *et al*.^43^). The location and an overview of the networks are described and illustrated in Table 1 and Fig. 2. In addition to the spatial distribution of stations, we also display the monthly mean air temperature climatology for each network to provide a first look at the general climate conditions and seasonal variability across cities (Fig. 6). Differences in deployment strategy in individual cities are clearly evident in Fig. 3, Fig. 4 to Fig. 5. It is also important to acknowledge the inherent differences between networks. No standard exists for urban meteorological networks and hence different approaches are common. Each network uses different (one or several) types of sensors, different installations, and configurations for collecting temperature data, leading to differences between networks. For instance, in Bern and Zurich, a large proportion of stations rely on self-built, low-cost devices (Gubler *et al*.^15^; Anet *et al*.^43^), while Novi Sad and Ghent utilize commercial sensors such as the ChipCap 2 and PT100 PRT probes, respectively (Šećerov *et al*.^38^; Caluwaerts *et al*.^35^). Ventilation strategies also vary: some sites use passively ventilated housings (e.g., Bern prior to 2023) while others employ actively ventilated shields (e.g., Ghent, see Supplementary Table 1). Additionally, the temporal coverage of the datasets varies substantially among networks (see Table 1). This variability reflects differences in project scope, duration, and instrumentation logistics and in some cases, sensor relocations, such as those in Bern, where devices moved due to construction activities or municipality requests. Shorter records (e.g., Bern, Biel) are associated with recent pilot or seasonal field campaigns. For example, the Bern network initially only measured in summer to record the strongest urban heat island effects during the warm season (Gubler *et al*.^15^). On the other hand, longer records (like Basel and Novi Sad) indicate permanent observation sites or continuous, multi-year monitoring operations. In several cases, such as the Ghent and Rennes network, data collection continues beyond the timeframe covered in the FAIRUrbTemp release. Users interested in more recent observations are encouraged to contact the respective network operators for access to extended datasets. Data storage formats and time references differ as well, some networks present their data in local time, others are in UTC. Concerning metadata, the location of the stations is reported in either a local coordinate system, the World Geodetic System 1984 (WGS84), or Universal Transverse Mercator (UTM). There are also inconsistencies in metadata IDs and station names across the networks. These data-metadata inconsistencies will become increasingly challenging when studying more than one network. It is important to note that each contributing network’s metadata practices directly influence the completeness and quality of the metadata in our repository. Because of this, there are still knowledge gaps that need to be filled in order to fully describe and compare measurements, especially when it comes to detailed instrument specifications and standardized best practices. The disparities in device type, cost, and setup undoubtedly influence the accuracy and comparability of temperature measurements. While this dataset does not yet include full instrumentation metadata for all 12 networks, it is clear that calibration protocols and sensor metadata play a critical role in ensuring data quality and should be documented wherever possible. Table 1 summarizes key information about the networks.

**Table 1:** Overview of the geographic and structural characteristics of the temperature-monitoring networks.

Network	Country	Lat/Lon	Sensor Type	Number of Stations	Sensor Height (m)	Period	Interval	References
Amsterdam	Netherlands	52.36, 4.90	VP-3; Decagon devices covered by a cylindrical shield from Davis Instruments	23	4 m	2014–2023	5 min	Ronda *et al*.^30^
Basel	Switzerland	47.55, 7.60	Pessl LoRain	217	3 m	2020–2022	15 min	Schlögl *et al*.^29^
Bern	Switzerland	46.95, 7.42	Hobo Pendant 8k	50-85	3 m	Summer 2019-2022	10 min	Gubler *et al*.^15^
Berlin	Germany	52.52, 13.40	Campbell Scientific CS215; Vaisala HMP155A; Pessl nMetos, …	11	2-3 m	2020–2023	5 min	Fenner *et al*.^36^
Biel	Switzerland	47.14, 7.25	Hobo Pendant 8k	40	3 m	Summer 2023	10 min	Erismann *et al*.^28^
Birmingham	England	52.59, -1.78	Aginova Sentinel Micro (ASM) and Vaisala WXT	23	2-3 m	2019–2022	5 min	Chapman *et al*.^60^, Müller *et al*.^11^
Freiburg	Germany	47.99, 7.84	Campbell Scientific ClimaVue50 and PESSL LoRAIN	44	3 m	2022–2023	1 min	Plein *et al*.^32^, Feigel *et al*.^33^
Ghent	Belgium	51.05, 3.73	PT100 PRT probe	6	2 m	2016–2023	1 h	Caluwaerts *et al*.^35^
Novi Sad	Serbia	45.26, 19.83	ChipCap 2 developed by GE Measurement & Control Co.	26	2-4 m	2014–2017	10 min	Šećerov *et al*.^38^
Rennes	France	48.11, -1.68	AWS Davis-VP2	23	2-3 m	2018	1 h	Dubreuil *et al*.^40^
Turku	Finland	60.45, 22.26	HOBO U23-001 HOBO MX2301A	67	3 m	2019–2021	30 min	Alvi *et al*.^42^
Zurich	Switzerland	47.39, 8.53	Sensirion SHT 31 Smart Gadget & Pessl LoRAIN v1	276	3 m	2019–2021	15 min	Anet *et al*.^43^

### Quality Control

Quality control is the process of detecting and labeling physically implausible or otherwise suspicious observations^44,45^. We devised a seven-step QC method to evaluate design flaws, communication failures, surrounding interruptions, or software errors. This is necessary to avoid possible errors within the datasets that could compromise the results of subsequent analysis^46^. It is acknowledged that some of the collected data has already undergone a QC, whereas other data has not (e.g. Plein *et al*.^32^). However, the further application of this QC method will ensure a minimum consistent level of QC process across all dataset. Our method combines two group of tests : (i) *physical-plausibility tests*, which apply on a single time series and reject values that are physically impossible or climatologically extremely unlikely, and (ii) *contextual tests*, which flag observations as suspicious when their spatial or temporal behavior significantly deviates from nearby values, but this is not necessarily impossible. Table 2 provides an overview of the seven tests (in each step, observations are assigned a flag of 0 or 1, referring to “non suspicious” or “suspicious”, respectively); an observation that fails any of the tests receives a flag of 1 in the final file.

**Table 2:** Summary of the seven quality control (QC) tests applied to the temperature time series.

QC Test	Description	Parameter	Value of parameter	Reference
L1. Gross errors	Report impossible values in the time series.	*T*_*a*_	${T}_{a,\min }=-40$^°^C ${T}_{a,\max }=60$^°^C	Dandrifosse *et al*.^47^
L2. Out of range	Find values that exceed user-selected climatological thresholds.	${T}_{a,\min }$ ${T}_{a,\max }$	For each city and season, the extreme values are defined. e.g. Freiburg (summer): ${T}_{a,\min }=1.72$^°^C ${T}_{a,\max }=41.90$^°^C	Hubbard *et al*.^61,62^
L3. Time consistency	Identifies data points whose change exceeds a defined limit.	${T}_{a,\min }^{C}$ ${T}_{a,\max }^{C}$	5-15 min: ± 3^°^C 30 min: ± 4^°^C Hourly: ± 4.5^°^C Daily: ± 20^°^C	WMO^51^, Vergauwen *et al*.^45^
L4. Temporal persistence	Report equal or near-equal values over three consecutive hours.	*T*_*a*_ ≠ *T*_*a*−1_ ≠ *T*_*a*−2_ ≠ *T*_*a*−3_ ≠ *T*_*a*−4_ ≠ *T*_*a*−5_ ≠ *T*_*a*−6_	*T*_*a*_ − *T*_*a*−1_ = 0 10 min: 36	Cerlini *et al*.^63^
L5. Climatic outliers	Flag values outside interquartile-range bounds.	ext_lim_factor	4	Brunet *et al*.^26^
L6. Spatial consistency	Compare each record to a weighted mean of neighboring stations.	sensitivity_scaling (*k*) min_abs_diff (*δ*)	*k* = 6 (general) *k* = 7 (dense urban cores) *δ* = 3	Shekhar *et al*.^64–66^
L7. Spatiotemporal consistency	Check for values implausible in space and time.	Radius Minimum neighbours	*lmt*_*xy*_ = 2500 m *lmt*_*n*_ = 5	Hamada *et al*.^67,68^

#### Physical plausibility tests

1. Gross errors: This step consists of the flagging of numerical values larger than 60 or lower than −40^°^C^47^. In this step, we flagged values such as −999, 125, or 98, which certain networks and systems have used or arise from sensor malfunctions, default device settings when no measurement is taken, or transmission failures.
2. Out of range: Observations were flagged when they exceeded physically plausible daily temperature extremes, defined by an extended regional climatological safety margin. Specifically, we used the ERA5-Land reanalysis dataset (1995–2023) to extract empirical maximum and minimum values for each study area. Given the lack of nearby official meteorological stations in some locations, ERA5-Land offered a consistent and spatially comprehensive alternative. This version of ERA5 is designed for land surface applications^48^. Its 9 km spatial resolution is finer than that of ERA5 and ERA-Interim, which are 31 km and 80 km, respectively. To address known cold biases in ERA5-Land temperature fields, especially during heat extremes^49,50^, We applied an additive correction of up to +6^°^C to the ERA5 air temperature data with the magnitude of the adjustment estimated from comparisons with reference station observations, and also partial consideration of the Urban Heat Island effects. The defined threshold was not intended to represent climatological normals (which are addressed in step 4), but rather to identify physically implausible outliers, such as unrealistically high values (>55^°^C in central Europe), and to ensure basic physical consistency across all networks. We emphasize that in future research, site-specific characteristics, particularly height and local land-surface variability, may call for more precise bias corrections (e.g., elevation-matched quantile mapping).
3. Time consistency: To ensure time series data stays consistent and reliable, we flagged values as potentially suspect when unexpected temperature changes occurred. For this evaluation, we used a median-based filter that compares each temperature observation with the median of its previous and subsequent values (within a ±3 time step window). If a data point deviated by more than a set threshold (±3^°^C for 5-minute to 15-minute data, ±4^°^C for 30-minutes, ±4.5/5^°^C for hourly data and ±20^°^C for daily data), it was flagged as a potential outlier. This approach, inspired by WMO guidance (WMO, 1993^51^, Beele *et al*.^52^ and Espinoza *et al*.^53^), ensures detection of abrupt and potentially erroneous changes while accounting for genuine atmospheric variability.
4. Climatic outlier: flagging of daily extreme (low and high) air temperature values based on statistical values. The statistical algorithm flags values that exceed the 1st or 3rd quartile by more than 4 times the interquartile range. This method was applied independently for each calendar month to account for the seasonal variation in temperature distributions. The threshold multiplier *M* = 4 was determined empirically in this study, after trying out different values on the datasets, aiming to catch real anomalies with the goal of minimizing false positives (flagging meteorologically valid extremes) while still identifying likely anomalies. There is no universal formula for picking M, but a value of 4 provides balance, catching problems without being overly reactive. This aligns with other QC methods that lean toward stricter thresholds when dealing with daily climate extremes.

#### Contextual tests (suspicious values)

1. Temporal persistence: The persistence test examines whether the same value has been recorded over an extended time period, indicating a potential sensor malfunction. Nonetheless, variability also depends on prevailing weather patterns; for example, winter low-stratus conditions might provide genuine sequences with minimal hourly temperature fluctuations. The test flags cases where the standard deviation over a moving 6-hour window is near zero in sub-hourly data, which is highly unlikely for a working sensor^54^. The test is flexible enough to handle missing data (up to 50% in a window) and only needs at least 5 valid readings to run. We acknowledge that this criterion may result in some false positives by mistakenly classifying valid observations as errors, especially during prolonged low-variability winter episodes.
2. Spatial consistency: In this test, each measurement ${x}_{i}^{t}$ at station *i* and time *t* is compared against a local spatial consensus formed from nearby stations within a 3 km radius, in a dynamic way which first selects the closest stations and then expand the radious to 3 km (Table 2). If even within 3 km there are too few stations, we allow the test to proceed when the effective number of neighbors ${n}_{e\mathrm{ff},i}^{t}={({\sum }_{j}{w}_{ij})}^{2}/{\sum }_{j}{w}_{ij}^{2}$ within 3 km is at least 1.5; otherwise the case is marked “insufficient information”. Neighbors are down-weighted by distance and further adjusted by land-use similarity (e.g., urban vs. vegetated). A reading is flagged if its deviation from the local consensus exceeds both a local statistical threshold and a minimum absolute difference *δ* (default *δ* = 3^°^C). This absolute floor *δ* (and the sensitivity multiplier *k*) is a variable in the shared code that users can change to fit their own needs; see github.com/StarAmini/QC_URBNET. This follows the standard definition of spatial outliers as values that deviate from their local neighborhood rather than the global distribution and uses well-established distance-based spatial weights. The weighted spatial mean ${\widehat{x}}_{i}^{t}$ of neighbouring stations is computed as:
  1 $${x}_{i}^{{\prime} t}=\frac{{\sum }_{j\ne i}{w}_{ij}\,{x}_{j}^{t}}{{\sum }_{j\ne i}{w}_{ij}}.$$
  Weights *w*_*ij*_ combine a Gaussian distance-decay kernel and land-use similarity and are capped at 3 km:
  2 $${w}_{ij}=\exp (-\frac{{d}_{ij}^{2}}{2{\sigma }_{d}^{2}})\,{\lambda }_{ij}\,{\bf{1}}\{{d}_{ij}\le 3000\,m\},$$
  where *d*_*ij*_ is the great-circle (Haversine) distance between *i* and *j*; *σ*_*d*_ is a distance bandwidth (set once per network; we use the network median pairwise distance); and *λ*_*ij*_ ∈ [0, 1] is a land-use similarity factor (e.g., 1.0 if identical land use; 0.4 if both vegetated/forested; 0 for highly dissimilar classes such as water vs. sealed/vegetated). The weighted local spread (computed over neighbors with *w*_*ij*_ > 0) is
  3 $${\sigma }_{i}^{{\prime} \,t}=\sqrt{\frac{{\sum }_{j\ne i}{w}_{ij}\,{({x}_{j}^{t}-{x}_{i}^{{\prime} t})}^{2}}{{\sum }_{j\ne i}{w}_{ij}}}.$$
  A value ${x}_{i}^{t}$ is flagged when
  4 $$| {x}_{i}^{t}-{x}_{i}^{{\prime} t}|  > \max (k\,\mathop{\mathop{\sigma }\limits^{{\prime} t}}\limits_{i},\,\delta ),$$
  with *k* a sensitivity multiplier (default *k* = 6) and *δ* the minimum absolute difference (default *δ* = 3^°^C, user-tunable). We require at least two valid neighbors within the adaptive radius or, failing that, an effective support of *n*_eff_ ≥ 1.5; at most the *K* nearest neighbors (default *K* = 5) are retained for stability. In our analyses we use a single global rule per network: the nearest valid neighbors within a hard cap of 3.0 km; require ≥2 neighbors or *n*_eff_ ≥ 1.5; use *δ* = 3^°^C (user-tunable), *k* = 6, retain up to *K* = 5 neighbors, and set *σ*_*d*_ to the network median pairwise distance.
3. Spatiotemporal consistency: This test flags outliers that are simultaneously extreme in both space and time. A temperature reading is flagged if it deviates significantly from the measurements of five nearby stations (located within 2.5 km) and from its own preceding and following time steps, exceeding the 99.99^th^ percentile of their respective distributions. Nearby stations are selected dynamically based on the location, and a flag is raised only when the reading is extreme in both spatial and temporal dimensions.

The sequence of QC checks is aligned with the steps outlined in the paper. Before each QC step is applied, the time series is updated to exclude the flagged values in the previous step. This approach minimizes the risk of errors carrying forward into later stages of analysis, ensuring robust and accurate results.

It is important to note that, initially, based on established references mentioned in Table 2, we modified the QC thresholds as necessary by applying our knowledge of the local climate and sensor behavior. In fact, we came across situations where a given threshold guidance was not sufficient or was not entirely relevant. We tested a range of parameter values across different cities to identify those that consistently flagged clear anomalies while avoiding the misclassification of normal, but unusual, weather patterns.

We note that there is room for improvement in the spatial consistency check. For example, the current method ignores the elevation effect, which should be taken into consideration for future improvements, as it may have a substantial impact on temperature variability.

Through a combination of literature and practical testing, we were able to develop a QC process that works reliably across our European datasets. Although it offers a strong foundation for urban temperature networks, it might require adjustment for environments outside of temperate our study areas.

The method only flags potentially suspicious values without touching the original data. It’s up to users to decide whether to remove or correct those values, depending on their own scientific judgment, as Brunet *et al*.^26^ recommend.

### Aggregating sub-hourly values to hourly and daily data

Given the different recording intervals used in the networks, the decision was taken to standardize recording intervals across the broader dataset. Here, we provide hourly averages, which are relevant to capture diurnal temperature variations^55^, urban heat island effects^56^, and human thermal comfort^57^. In addition, we provide daily maximum and minimum temperatures, which are widely used diagnostics for understanding climate trends and extreme weather events. Therefore, we aggregated sub-hourly into hourly means and extracted daily maxima and minima^20^. The aggregated values are set to missing if: less than 80% of sub-hourly data are available per hour (for hourly data) or per day (for daily max/min values)^58^.

Data aggregation in the hourly step was applied to the raw data. Afterwards, our QC method was applied to both the raw data and the hourly averages. However, to create daily data, we used quality-controlled data in their original time step, meaning that flagged sub-hourly values were excluded from the calculation.

## Data Records

The final FAIRUrbTemp dataset is provided in the Station Exchange Format (SEF), a standard format for the exchange of meteorological data defined by the Copernicus Climate Change Service^26^. It consists of one metadata file as a compressed folder (.zip), a readme file as a text file (.tsv), and 12 compressed folders (.zip). Each of these folders relates to one of the 12 studied cities and includes 5 subfolders. To ensure consistency across the diverse contributing networks, all time information has been converted to UTC, and all geographic coordinates have been harmonized to the WGS84 reference system. Data are publicly accessible through the BORIS Portal of the University of Bern: 10.48620/93247^59^.

- RAW: Text files (.tsv) for each station that include the near-surface temperature data shared by the project partner in a raw format before applying any QC checks from our side. Each file begins with the station metadata and ends with the temperature time series. The Metadata include: station code (*ID*), which follows the country_city_stationcode convention (e.g., for station code 2195 in Amsterdam, Netherlands, the *ID* is “*NLD_AMS_2195*”); station name (*Name*) using the same convention; latitude in decimal degrees (*LAT*); longitude in decimal degrees (*LON*); Altitude in meter (*ALTs*); the center that shared the data (*Source*); a link to the network dataset, if available (*Link*); the measured variable (street-level temperature) (*Vbl*); time statistics (point(state), average, min/max) (*Stat*); unit of the measured variable (*Units*); metadata (*Meta*). The second section at the bottom of the text files contains the time series dates. The column (*Period*) indicates the state of the time statistics; if it is 0, the record is point data. Temperature values (*Value*) and (*Meta*) include additional metadata or descriptions.
- QC: Text files (.tsv) for each station that include the near-surface temperature data checked with our QC method. The file format is identical to the previous version; the only difference is that the column (*Meta*) indicates which QC test flagged the data.
- Hourly data: Text files (.tsv) for each station that include nearsurface temperature data aggregated to hourly timestamps. Results of the QC check are also documented in the (*Meta*) column.
- DailyMax: Text files (.tsv) for each station with the daily maximum temperature, computed from the QCchecked data.
- DailyMin: Text files (.tsv) for each station with the daily minimum temperature, computed from the QCchecked data.

For clarity in the *Meta* column, suspicious values detected by the QC process are marked with the QC test that identified the issue, denoted by the prefix “*qc* = ” in the “Meta” column. For example, “*qc* = *temporal*_*coherence*” indicates a failure in the temporal coherence test, signalling that the associated value should be treated as unreliable for most analytical purposes.

The final metadata file (.zip) has 12 subfolders for each of the 12 cities. In each city’s folder, the Metadata is structured at three levels:

- Station-level metadata includes city name (*city*); station ID (*station id*); station number (*station_number*), latitude in decimal degrees (*LAT*); longitude in decimal degrees (*LON*); and sensor height in meter (*sensor_height_m*). It is worth mentioning that the header of the data files for a station contains even more detailed metadata, such as data source and links to the original network’s webpage.
- Measurement-level metadata consists of city name (*city*); station ID (*station id*); measurement interval (*measurement_interval*); sensor type (*sensor_type*); measured variable (*measured_variable*); units (*units*); and the type of QC tests which flag the data in the station (*qc_flag*).
- Network-level metadata provides information about each contributing urban network, including network name (*network_name*); geographic coverage (*geographic_coverage*); operator or owner (*operator or owner*); funding source (*funding_source*); number of stations (*number_of_stations*); measurement parameters (*measurement_parameters*); measurement interval (*measurement_intervals*); statistical methods (e.g., point or average measurements) (*time_statstics*); data format (*data_format*); accessibility (*accessibility*); and contact details (*contact_details*).

While the standardized metadata provide a consistent and comparable overview of the dataset, they are not a substitute for more detailed site-specific documentation (e.g., maps, photographs, and skyview factor estimate), as recommended by Oke (2004, 2017). For selected station networks, some additional documentation is available in Table 1 in the supplementary material.

## Technical Validation

In this section, we have evaluated the QC approach applied to the FAIRUrbTemp dataset. We begin by presenting the results of the QC procedures, followed by statistical evaluations of the effect of the sensors and land cover on the quality of the measured data.

### Quality control

After applying the QC procedure described in the Method section, we finally obtained a total of 809 quality-controlled station series. The QC analysis, summarized in Table 3, highlights the proportion of flagged data across various cities, providing a benchmark for overall data integrity. Note that not all networks use the same sensor types, and some have already undergone initial checks.

**Table 3:** Counts and percentages of measurements flagged by each QC test, for each network.

Network	Measured data	Number of Flagged Data						
		gross_errors	out_of range	time consistency	temporal persistence	climatic outliers	spatial consistency	spatiotemporal consistency
**Amsterdam**	23 196 696	83 138 [0.36%]	200 [0.001%]	23 [~0%]	34 627 [0.15%]	71 [~0%]	23192 [0.1%]	1 [~0%]
**Bern**	3 920 400	0	0	6 [~0%]	0	0	4 570 [0.12%]	139 [0.003%]
**Basel**	22 832 740	0	0	54 [~0%]	52 827 [0.23%]	1 268 [0.006%]	1 530 [~0.007%]	4 [~0%]
**Biel**	714 000	0	0	16 [0.002%]	1 512 [0.21%]	0	1 174 [0.16%]	0
**Birmingham**	6 845 881	607 [0.009%]	481 [0.007%]	30 [~0%]	0	90 [0.001%]	1 421 [0.021%]	0
**Freiburg**	40 302 328	41 761 [0.104%]	2 [~0%]	142 [~0%]	0	0	322 [0.001%]	0
**Ghent**	382 950	0	0	9 [0.002%]	0	0	0	0
**Berlin**	4 631 561	0	0	0	86 [0.002%]	0	0	0
**Novi Sad**	5 614 466	291 554 [5.19%]	2 186 [0.039%]	2 106 [0.038%]	23 [~0%]	84 [0.001%]	5 766 [0.103%]	99 [0.002%]
**Rennes**	202 078	0	11 [0.005%]	0	0	1 [0.002%]	17 [0.008%]	0
**Turku**	3 524 870	0	0	1 [~0%]	54 [0.001%]	0	85 [0.002%]	0
**Zurich**	24 188 916	9 956 [0.041%]	1 033 [0.004%]	474 [0.002%]	30 [~0%]	718 [0.003%]	45 689 [0.07%]	2 [~0%]

Across the full multi-city dataset (about 1.36 × 10^8^ individual records), less than 0.5 % of all measurements were flagged by any single test, indicating overall good data quality before QC. The majority of QC flags were triggered by inconsistencies in the gross error check. This test flags about 0.31 % of all measurements, effectively removing obviously invalid codes and corrupted readings. The remaining physical checks (out of range, temporal consistency and climatic outlier tests) each affect significantly less than 0.01 % of all observations, confirming that values which are physically or climatologically implausible are relatively rare in the raw dataset. The second and third largest shares of flagged observations were associated with the spatial consistency test (0.08 %), and the temporal persistence test (0.06 %). The spatiotemporal consistency test has only a very minor impact at the network scale (well below 0.01 %). Put together, these contextual checks add a conservative layer to the physical plausibility assessment by highlighting observations whose behavior deviates from their temporal or spatial neighborhood.

For the majority of networks, the overall fraction of flagged measurements remains below 0.3 %. For instance, rejection rates in Ghent and Birmingham are less than 0.03 % in all tests, indicating very stable sensor behavior or serious prior screening. In several networks (Bern and Zurich), the spatial consistency test is the dominant source of contextual flags, but even there it typically affects less than 0.1–0.2 % of the local measurements.

On the other side, two networks stand out with somewhat higher fractions of flagged data and illustrate different QC behaviours. In Amsterdam, about 0.75 % of all values are flagged, mainly due to a combination of physical gross errors (0.36 %) and contextual temporal-persistence (0.30 %) and spatial-consistency (0.09 %) flags. The Novi Sad network exhibits the largest fraction of flagged data, with approximately 5.3 % of all measurements marked as problematic; this is almost entirely driven by the gross-error test (5.19 %), pointing to a large number of clearly invalid readings that are effectively removed by the physical plausibility screening.

#### Expert-based confusion matrix evaluation

To validate how well our automated QC distinguishes between problematic and acceptable data, we complemented the flag statistics with an expert-based confusion-matrix evaluation. Since no independent “ground truth” reference exists for sub-hourly, street-level air temperature, we used local expert judgment as the best available proxy. For four networks,140 measurments were randomly selected, including 70 that had been flagged at least once by the QC, and 70 that had never been flagged.

For each network, a local expert manually examined the full temperature time series and classified each data point as either “problematic” (containing clearly erroneous or systematically biased records) or “acceptable” (measurements judged physically plausible). These expert labels were then compared to the binary QC outcome (flagged vs. not flagged) to construct confusion matrices at the station level (Table 4). In the confusion matrix analysis, we defined the positive class as data points containing problematic or erroneous records, and the negative class as points with acceptable records. Accordingly, a QC flag corresponds to a predicted measurement (problem detected), whereas an unflagged measurement corresponds to a predicted negative (no problem detected).

**Table 4:** Expert-based evaluation of the automated QC decision for three networks.

Network	TP	FP	TN	FN	Accuracy (§%)	Precision (%)	Specificity (%)
Novi Sad	64	6	70	0	95.7	91.4	92.1
Zurich	68	2	65	5	95.0	97.1	97.0
Amsterdam	37	23	70	0	76.4	61.7	75.3

TP = true positives; FP = false positives; TN = true negatives; FN = false negatives. Metrics are calculated relative to the 140 evaluated stations per network.

The results show that the QC system consistently minimizes missed errors across networks. In Novi Sad, the QC achieved high accuracy (95.7 %), precision (91.4 %), and specificity (92.1 %), with no problematic stations left undetected. In Zurich, the QC achieved high overall performance, with a recall of 93.2 % and a precision of 97.1 %. Only 7 % of problematic stations were missed by the QC, and less than 3 % of acceptable stations were incorrectly flagged. The few missed problematic cases show that, in rare cases, small sensor flaws might not appear as strong spatial or temporal differences.

Amsterdam represents a third case, where the QC achieved moderate precision (61.7%) and high recall (100%). The temporal-persistence test in Amsterdam produced the majority of false positives during long periods of weak winds, heavy cloud cover, and little daily temperature variation, especially in winter or during warm-front passages. Under such conditions, long intervals of nearly constant temperature are meteorologically plausible but can resemble sensor stagnation, suggesting that persistence thresholds may benefit from seasonal or diurnal adaptation.

Overall, the expert evaluation shows that while variations in false-positive rates reflect different urban and climatic settings, the QC system reliably minimizes missed errors across networks. These findings demonstrate the efficacy of the physical-plausibility checks and identify contextual assessments as a crucial area for additional improvement.

### Impact of Sensors

As mentioned in Table 1, networks may use a mix of different temperature sensors, and this does have potential impacts on the overall quality of the measurement. In order to investigate this further, we have piloted the Zurich network to determine the effects of this potential challenge. During the operational period of the network (2019–2021), two types of sensors (Pessl LoRAIN and Sensirion SUHIRS) were used. Thus, we ran QC checks in two ways: first, considering all sensors as one unified network and second, dividing the sensors into two groups based on their sensor type and checking each group separately (Table 5). Because the number of SUHIRS stations was nearly twice that of LoRAIN, the comparison of flagged data must be based on relative proportions rather than absolute counts. Once normalized to the total number of measurements, the results reveal clear differences between the two sensor types. Whereas, SUHIRS benefited from better radiation shielding and generally provided more trustworthy measurements under both day- and nighttime conditions. SUHIRS showed more out-of-range values and it is consistent with calibration artefacts during deployment, as SUHIRS underwent a 40^°^C-0 ^°^C calibration, which may explain anomalous values at the beginning of their records. For most other tests, the flagged fractions were smaller compared to the LoRAIN sensors. LoRAIN sensors showed a slightly higher percentage of gross errors overall and are prone to significant radiation biases of up to 6 K, as previously reported^43^.

**Table 5:** Number and percentage of flagged data at each QC step for two different sensor types of the Zurich Network.

Network	Number of Stations/points	Number of Flagged Data						
		gross_errors	out_of range	time consistency	temporal persistence	climatic outliers	spatial consistency	spatiotemporal consistency
**Zurich_combined**	276 (24 188 916)	9 956	1033	747	30	718	45 689	2
**Only SUHIRS sensors**	182 (15 950 662)	9 263 [0.058%]	943 [0.006%]	306 [0.002%]	0	401 [0.002%]	29490 [0.185%]	1 [~0%]
**Only LoRAIN sensors**	94 (8238254)	693 [0.084%]	90 [0.001%]	168 [0.002%]	30 [~0%]	317 [0.004%]	16 199 [0.197%]	1 [~0%]

**Fig. 7:**
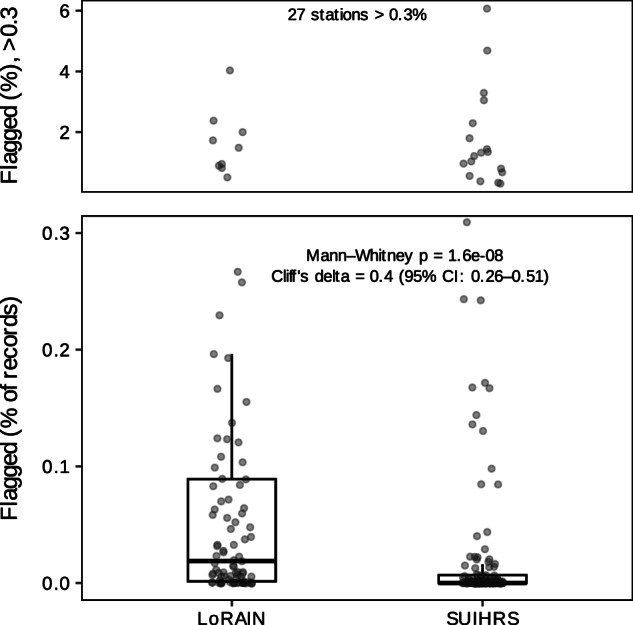
Impact of sensor type on data flagging in the spatial-consistency assessment across the Zurich network. Box plots show the per-station fraction of flagged temperature observations for Sensirion SUIHRS and LoRAIN sensors. Individual points represent stations. The lower panel highlights the main range of flagged fractions (0 −0.5%), while the upper panel shows stations exceeding 0.5%.

In the case of the spatial consistency check, Sensirion SUHIRS stations showed a significantly higher proportion of flagged observations compared to LoRAIN. Even though the total number of flags in this test appears lower in Table 5, the per-station statistical comparison shows the opposite pattern; SUHIRS stations have a significantly higher flag rate (Mann-Whitney *p* = 1.9 × 10^−8^; Cliff’s *δ* = 0.39, 95% CI: 0.26-0.51) (Fig. 7). This indicates that inconsistencies in SUHIRS are widespread among the stations, whereas LoRAIN issues are more concentrated in a limited number of sensors.

**Fig. 8:**
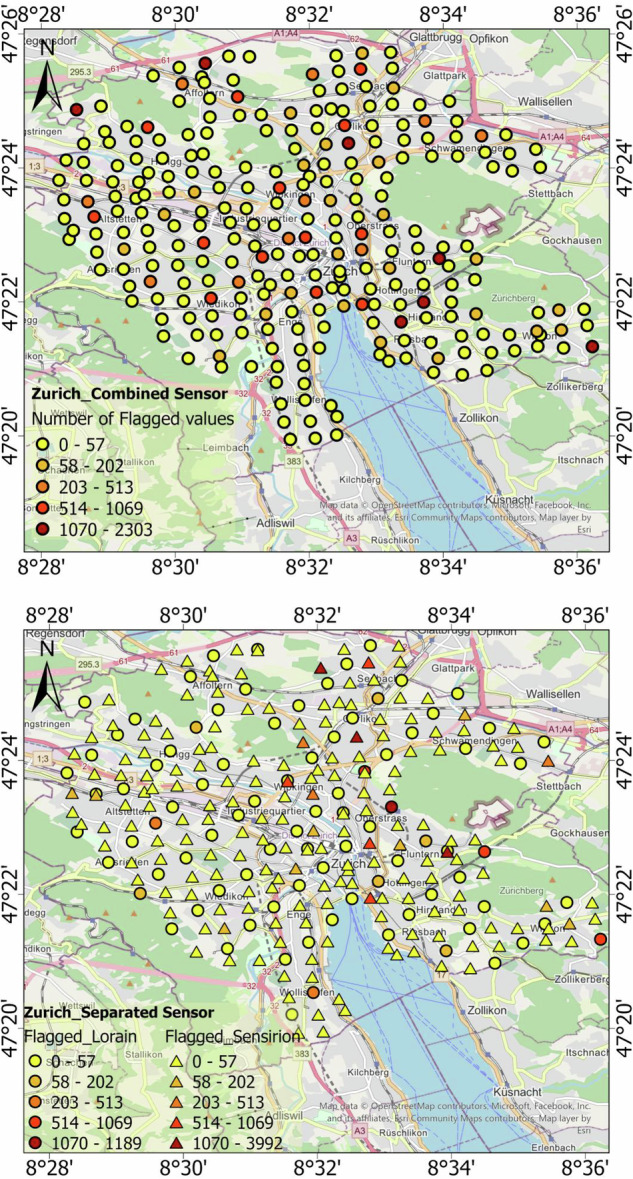
Spatial distribution of flagged data after applying spatial consistency check on the near-surface air temperature network of Zurich. (**a**) shows results when all sensors are checked together as one combined network, while (**b**) presents results after dividing sensors into two types: LoRAIN sensors (circles) and Sensirion SUHIRS sensors (triangles).

On the other side, when both sensor types were treated as one network (Fig. 8a), clusters of high flag counts appeared in areas dominated by SUHIRS, which also had an impact on the QC of nearby LoRAIN stations. After separating the sensor types (Fig. 8b), it became clear that the two sensor types interfered with each other, and that a large proportion of the problematic data originated from SUHIRS sensors, particularly during the pre-deployment calibration period. These results (Table 5) clearly indicate that differences between sensor types significantly affect the data quality and the reliability of spatial consistency checks. While separating sensors resolves cross-interference, the analysis also showed that the large radiation errors affecting LoRAIN stations^43^ were not fully detected by the current algorithm. To address this limitation, integrating cross-validation with nearby reference stations from the local weather service would be beneficial in future work.

Overall, these results demonstrate that sensor type has a measurable impact on near-surface air temperature data quality. Differences in radiation shielding, calibration procedures, and sensor response characteristics influence the frequency and type of QC flags and should be explicitly considered when interpreting dense urban temperature observations.

### Impact of Land Cover

**Fig. 9:**
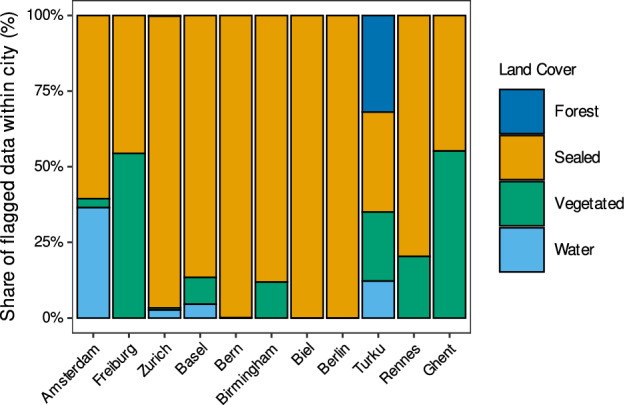
Fractional impact of Land cover types on QC flagged across the studied weather networks. Each bar shows the normalized share (100%) of flagged temperature observations within a city.

To evaluate the effect of different land covers on the performance of temperature measurements, we classified land cover into four classes (sealed, vegetated, forest, and water) and calculated the proportion of QC flags within each land cover type across the studied networks. The results show that there is a marked difference between each land cover class (Fig. 9). The number of relative flag rates exhibited a high value for sealed areas compared to vegetated, forest, and water bodies (Kruskal–Wallis, *p* < 0.001; Cliff’s *δ* = 0.37). While this partly reflects increased sensor exposure to anthropogenic influences, it is also linked to enhanced microclimatic variability in urban environments. In particular, local effects such as reflected shortwave radiation from parked vehicles, building facades, or paved surfaces can transiently heat sensors and produce sharp local temperature contrasts. This pattern continued after normalization by the number of stations and total observations, which showed that it was not caused and biased by uneven sampling density. The findings show a significant dependency of the spatial and temporal consistency checks on land cover, which is a sign of more microclimatic variability in built-up areas. Overall, the result demonstrates that local land cover can considerably affect the street-level temperature data quality, especially in dense urban networks.

To examine the impact of land cover on one of the most extensively flagged tests in depth, we incorporate the classical spatial-consistency check by integrating land cover similarity into the neighbor weighting scheme. Instead of relying solely on geographic proximity, we upweight comparisons between sensors in similar land covers. This means that nearby stations on the same land cover are comparable with each other, while those on very different classes (such as water versus urban pavement) are not comparable. Otherwise, each neighbor contributes equally when land cover is ignored, and actual temperature differences driven by physiography often appear as false positive outlier flags. To show this effect, we applied both approaches to the studied networks. The results show that considering the land cover classes in this QC check decreases the number of flagged values in all networks. This reduction in Amsterdam, Bern, Basel, Biel, Freiburg, Novi Sad, Rennes, Turku, Zurich is 2.9, 63, 94, 74, 60, 12, 99, 98 and 54 percent respectively. However, in Birmingham and Berlin, both cases stayed zero, and the check has not been applied to the Ghent dataset because of the existence of just 6 stations.

**Fig. 10:**
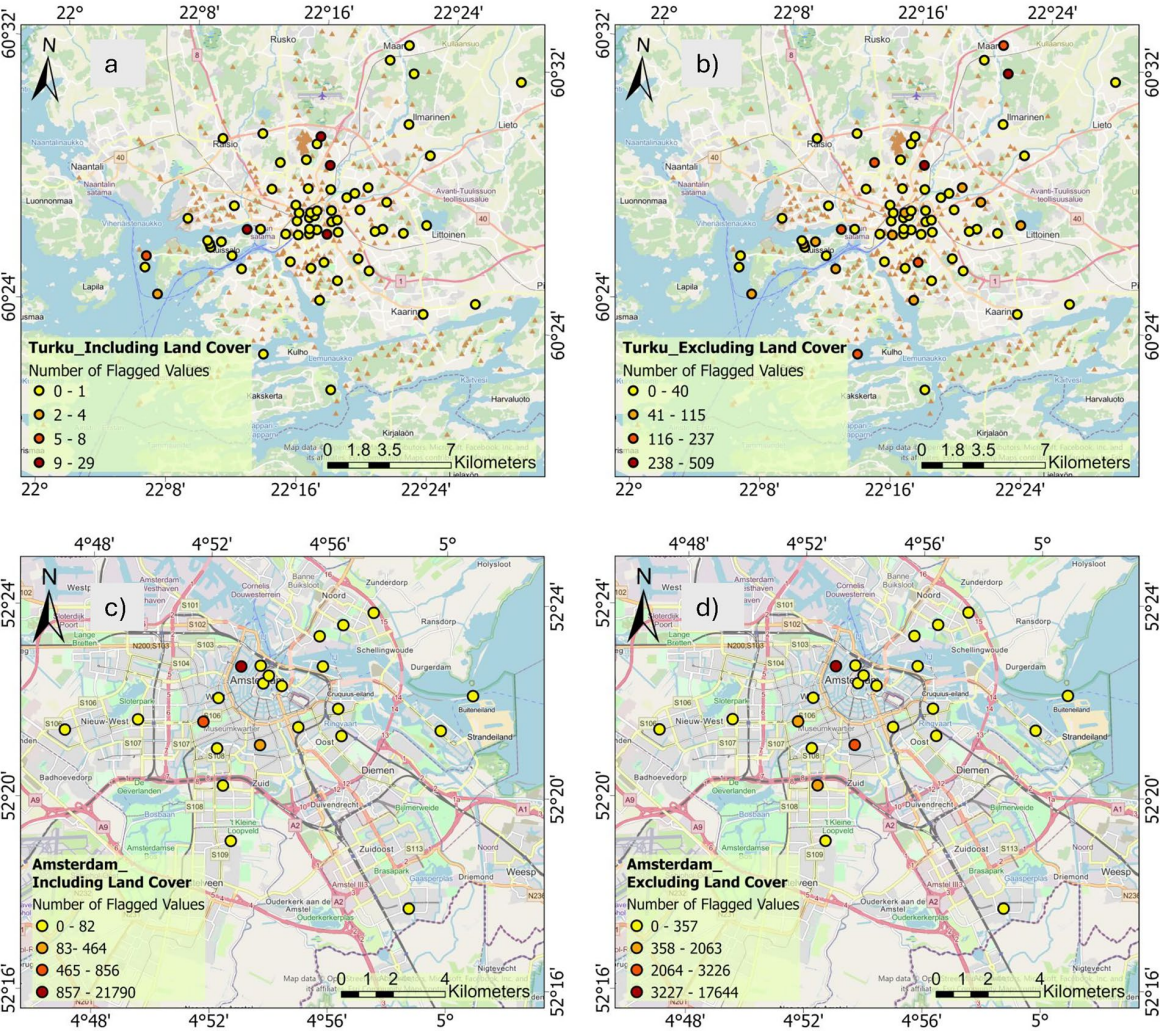
Comparison of the spatial-consistency check results in the network of Turku, (**a**) excluding the land cover effect from the check) and (**b**) including the effect of land cover in the check. The second row shows the network of Amsterdam, (**c**) excluding the land cover from the check, and (**d**) considering the land cover in the check.

We chose two representative case studies to clearly demonstrate these disparate effects: Amsterdam, which had one of the smallest reductions (2.9%), and Turku, which had one of the largest reductions (98%) (Fig. 10). In Turku, a city characterized by pronounced coastal-inland gradients and heterogeneous land cover, many sensors placed near water bodies were initially flagged as inconsistent due to their temperature differences from inland urban stations. Incorporating land-use weighting reduced these false positives substantially, as genuine environmental contrasts were recognized rather than flagged as errors. Adding land-use weighting reduced these false positives a lot because genuine differences in the environment were found instead of being marked as mistakes. Conversely, Amsterdam exhibits a more uniform urban environment with mostly sealed surfaces and fewer sharp environmental gradients. So, the unweighted spatial-consistency test already flagged a small number of stations, and adding land cover data offered minimal additional benefit, reflecting the city’s homogenous urban landscape.

In conclusion, integrating land-use weighting into the spatial consistency QC check effectively reduces false positives, resulting in a cleaner and more reliable dataset. However, local environmental factors have a significant impact on QC outcomes; as a result, to validate the dataset and make sure that data interpretations accurately reflect local environmental conditions, we advise involving local expert insights prior to practical use.

Overall, the results demonstrate that the applied QC framework effectively identifies erroneous and suspicious measurements while preserving the majority of physically plausible observations. Differences in flag rates between networks are largely explained by variations in sensor characteristics, topography, land cover, and prevailing meteorological conditions, rather than systematic deficiencies in the QC procedure. While contextual tests may be conservative in highly heterogeneous or weakly forced urban environments, they provide an important safeguard against undetected errors. Taken together, the FAIRUrbTemp dataset offers a quality-controlled, multi-city collection of street-level air temperature observations, providing an important source for studying urban climate variability and thermal processes across European cities.

## Usage Notes

The UrbFairTemp dataset is a highly valuable resource in Europe, with broad applications in fields like climate science, health, and urban planning. For the first time, it brings together the near-surface air temperature time series from urban meteorological networks in a consistent format, making the analysis process significantly more efficient. Additionally, it provides comprehensive access to raw sub-hourly data, as well as quality- controlled, hourly, and daily data.

The quality control method is derived from best practices used in existing studies and allows an evaluation of the data from various perspectives. It is also adaptable to different datasets, even those with their own inherent errors. However, it is important to note that the applied quality control approach is automated and robust, focusing on the detection of outliers and suspicious measurements rather than on physical error correction. Hence, further developments are still needed in consistent quality control strategies, particularly regarding the integration of physically based corrections, such as radiation effects. This is particularly crucial for low-cost measurement devices. These devices are prone to errors, especially in daytime data, due to radiative influences^43^.

In order to define empirical maximum and minimum temperature values, we used ERA5 Land data, which are freely available and easy to obtain. However, other datasets could be used, and in future studies, this could be investigated.

It is essential to acknowledge that FAIRUrbTemp compiles harmonized data from selected research-oriented networks that are members of the consortium rather than attempting to provide a comprehensive inventory of every urban weather station in every city. In some cities, additional urban meteorological stations are operated by other institutions; for instance, in Berlin, networks run by the German Weather Service (DWD), the Freie Universität Berlin, and the city administration are available via the external platform uco.berlin, but are not included in FAIRUrbTemp. These initiatives highlight the importance of coordinated data sharing for improving spatial coverage, and long-term usability of urban climate observations.

Lastly, we should emphasize that there are currently no plans to update FAIRUrbTemp. However, since this dataset is developed under the COST Action project FAIRNESS (CA20108), several related initiatives are underway. In line with its objectives, COST Action FAIRNESS, for example, seeks to offer high-quality data in every European country. It also outlines policies and strategies for data collection and the establishment of observational networks. The quality control schema and principles outlined in this paper are also considered to be broadly applicable. For people who just read the abstract and usage notes, we would mention at least here, if not in both places, that the quality control never excludes/deletes any data but just adds flags. And that the user needs to filter the data to their needs.

## Supplementary information

Supplementary file

## Data Availability

All data used in this study is publicly accessible online under the CC-BY licence via the following links: 10.48620/93247.

## Code availability

The data processing and QC routines are written in R (v.4.3.1) programming language. The entire code used is freely available at GitHub (https://github.com/StarAmini/QC_URBNET) under the GNU General Public License v3.0.
