# Supplementary material for: Comprehensive compilation and quality assessment of street-level urban air temperature measurements across European networks
Source: Sci Data. 2026 Feb 14;13:658. doi: 10.1038/s41597-026-06804-4 (PMC13121598; doi:10.1038/s41597-026-06804-4)
Supplement: Supplementary file 1 — Supplementary file [file 41597_2026_6804_MOESM1_ESM.pdf]

Supplementary Information for "Comprehensive compilation and quality assessment of street-level urban air temperature measurements across European networks"

Setareh Amini[1,2*], Adrian Huerta[1,2], Jörg Franke[1,2], Yuri Brugnara[1,2], Steven Caluwaerts[3,4], Julien Anet[5], Stevan Savić[6,7], Moritz Gubler[1,2,8], Gert-Jan Steeneveld[9], Lee Chapman[10], Fred Meier[11], Vincent Dubreuil[12], Andreas Christen[13], Matthias Zeeman[13], Branislava Lalić[14], Sebastian Schlögl[15], Jukka Käyhkö[16], AmirMasoud Azadfar[17], and Stefan Brönnimann[1,2]

[1]Oeschger Centre for Climate Change Research (OCCR), University of Bern, Bern, Switzerland
[2]Institute of Geography, University of Bern, Bern, Switzerland
[3]Department of Physics and Astronomy, Ghent University, Krijgslaan 281, 9000 Gent, Belgium
[4]Meteorological Institute of Belgium, Ringlaan 3, 1180 Ukkel, Belgium
[5]Umwelt- und Gesundheitsschutz (UGZ), Fachbereich Stadtklima, Zürich, Switzerland
[6]Faculty of Sciences, University of Novi Sad, Trg Dositeja Obradovica 3, 21000 Novi Sad, Serbia
[7]Faculty of Natural Sciences and Mathematics, University of Banja Luka, Mladena Stojanovica 2, 78000 Banja Luka, Bosnia & Herzegovina
[8]Institute for Lower Secondary Education, Bern University of Teacher Education, Bern, Switzerland
[9]Wageningen University, Meteorology and Air Quality Section, Wageningen, The Netherlands
[10]Iniversity of Birmingham, Edgbaston, Birmingham B15 2TT, UK.
[11]Institue of Ecology, Technische Universität Berlin, Berlin, Germany
[12]LETG-UMR 6554, CNRS, University of Rennes 2, F-35000 Rennes, France
[13]Albert-Ludwigs-Universität Freiburg, Environmental Meteorology, Freiburg, Germany
[14]Faculty of Agriculture, University of Novi Sad, Serbia
[15]meteoblue AG, Basel, Switzerland
[16]University of Turku, Department of Geography and Geology, Finland
[17]Department of Computer Science, Lakehead University, Canada
[*]corresponding author: Setareh Amini (setareh.amini@unibe.ch)

Network	Country	Lat/Lon	Sensor Type	Number of Stations	Sensor Height (m)	Study Period	Active Ventilation	Measurement Interval	Time statistics (state/avg/min/max)	Reference
**Amsterdam**	Netherlands	52.36, 4.90	VP-3; Decagon devices covered by a cylindrical shield from Davis Instruments	23	4 m	2014–2023	Y	state	5 min	Ronda et al.[?]
**Basel**	Switzerland	47.55, 7.60	Pessl LoRain	217	3 m	2020–2022	N	state	15 min	Schlögl et al.[?]
**Bern**	Switzerland	46.95, 7.42	Hobo Pendant 8k	50-85	3 m	Summer 2019–2022	N	state	10 min	Gubler et al.[?]
**Berlin**	Germany	52.52, 13.40	Campbell Scientific CS215; Vaisala HMP155A; Pessl nMetos, . . .	11	2–3 m	2020–2023	Y (CS215) N (nMetos)	avg	5 min	Fenner et al.[?]
**Biel**	Switzerland	47.14, 7.25	Hobo Pendant 8k	40	3 m	Summer 2023	N	state	10 min	Erismann et al.[?]
**Birmingham**	England	52.59, –1.78	Aginova Sentinel Micro (ASM) and Vaisala WXT	23	2–3 m	2019–2022	N	avg	5 min	Chapman et al. [?], Müller et al.[?]
**Freiburg**	Germany	47.99, 7.84	Campbell Scientific ClimaVue50 and PESSL LoRAIN	42	3 m	2022–2023	Y	state	1 min	Plein et al.[?], Feigel et al.[?]
**Ghent**	Belgium	51.05, 3.73	PT100 PRT probe	6	2 m	2016–2023	Y	hourly sampling from 1 minute data	1 h	Caluwaerts et al.[?]
**Novi Sad**	Serbia	45.26, 19.83	ChipCap 2 developed by GE Measurement & Control Co.	26	2–4 m	2014–2017	Y	avg	10 min	Šećerov et al.[?]
**Rennes**	France	48.11, –1.68	AWS Davis-VP2	23	2–3 m	2018	N	state	1 h	Dubreuil et al.[?]
**Turku**	Finland	60.45, 22.26	HOBO U23-001 HOBO MX2301A	67	3 m	2019–2021	N	state	30 min	Alvi et al.[?]
**Zurich**	Switzerland	47.39, 8.53	Sensirion SHT 31 Smart Gadget & Pessl LoRAIN v1	276	3 m	2019–2021	Y	state	15 min	Anet et al.[?]

Supplementary Table 1. Overview of the geographic and structural characteristics of the temperature monitoring networks.
